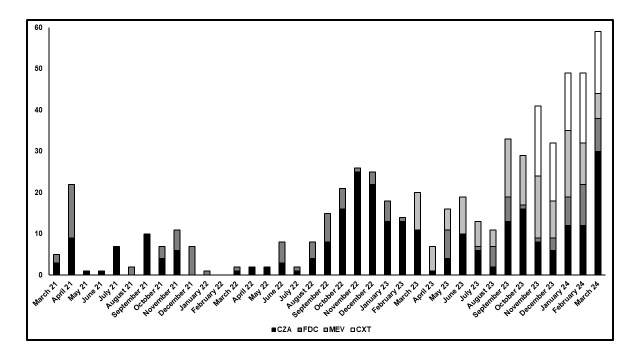# Utility of a TDM-Guided Expert Clinical Pharmacological Advice Program for Optimizing the Use of Novel Beta-Lactam/Beta-Lactamase Inhibitor Combinations and Cefiderocol in a Tertiary University Hospital: An Interim Analysis

**DOI:** 10.1097/FTD.0000000000001334

**Published:** 2025-05-02

**Authors:** Milo Gatti, Pier Giorgio Cojutti, Matteo Rinaldi, Simone Ambretti, Maddalena Giannella, Pierluigi Viale, Federico Pea

**Affiliations:** * Department of Medical and Surgical Sciences, Alma Mater Studiorum University of Bologna, Bologna, Italy;; † Clinical Pharmacology Unit, Department for Integrated Infectious Risk Management, IRCCS Azienda Ospedaliero-Universitaria di Bologna, Bologna, Italy;; ‡ Infectious Diseases Unit, Department for Integrated Infectious Risk Management, IRCCS Azienda Ospedaliero-Universitaria di Bologna, Bologna, Italy; and; § Operative Unit of Microbiology, Department for Integrated Infectious Risk Management, IRCCS Azienda Ospedaliero-Universitaria di Bologna, Bologna, Italy.

**Keywords:** expert clinical pharmacological advice program, personalized antimicrobial therapy, novel BL/BLIc, cefiderocol, multidrug-resistant gram-negative pathogens

## Abstract

**Background:**

This study assessed the utility of a therapeutic drug monitoring (TDM)-guided expert clinical pharmacological advice (ECPA) program to optimize aggressive pharmacokinetic/pharmacodynamic (PK/PD) target attainment of novel beta-lactam/beta-lactamase inhibitor (BL/BLIc) combinations and cefiderocol.

**Methods:**

All hospitalized patients who received TDM-guided ECPA with BL/BLIc (ceftazidime–avibactam, ceftolozane–tazobactam, or meropenem–vaborbactam) or cefiderocol were assessed retrospectively. Three performance indicators were identified: the average number of ECPAs delivered per month of availability of the program and the ratio between the total number of ECPAs recommending dosing adjustment and the total number of ECPAs, at the first and at subsequent TDM assessments. The relationships between aggressive PK/PD target attainment and clinical and microbiological outcomes were assessed.

**Results:**

A total of 595 ECPAs were administered to 263 patients to optimize 319 treatment courses. Novel agents were mostly used for targeted therapy (79.6%) by continuous infusion (CI; 82.8%). In the first TDM assessment, dose increases were mostly required for patients receiving intermittent/extended infusion (II/EI) (51.9% vs. 6.4%; *P* < 0.0001), whereas dose decreases were mostly recommended for patients receiving CI (60.3% vs. 23.1%; *P* < 0.001). In subsequent TDM assessments, the overall proportion of ECPAs recommending dosing adjustments decreased in both groups (57.1% and 39.3% in the II/EI and CI groups, respectively). Aggressive PK/PD target attainment was associated with the highest microbiological eradication rate for ceftazidime–avibactam (79.6% out of 86.0%; *P* < 0.001), and the highest clinical cure rate for ceftazidime–avibactam (64.2% out of 78.1%; *P* < 0.001) and cefiderocol (50.0% out of 51.5%; *P* = 0.006).

**Conclusions:**

A dedicated TDM-guided ECPA program may be helpful for optimizing the use of novel agents in settings with a high prevalence of multidrug-resistant pathogens.

## Abstract 2

Supplemental Digital Content is Available in the Text.

## INTRODUCTION

The global spread of difficult-to-treat resistant (DTR) gram-negative pathogens emerged as a critical health issue in the last decade, with remarkable morbidity and mortality in critically ill patients.^1^ Some novel beta-lactam/beta-lactamase inhibitor combinations (BL/BLIc), namely ceftolozane–tazobactam, ceftazidime–avibactam, meropenem–vaborbactam, and imipenem–relebactam, and a novel siderophore beta-lactam, namely cefiderocol, were recently approved for effectively counteracting this worrisome scenario.^2^

A multidisciplinary international position paper recently stated that therapeutic drug monitoring (TDM) should be considered the only safe and effective way for using antibiotics in critically ill patients.^3^ A recent meta-analysis showed that the TDM-guided approach of traditional beta-lactams in critically ill patients leads to either a higher likelihood of optimal pharmacokinetic/pharmacodynamic (PK/PD) target attainment or better clinical cure and microbiological eradication rates compared with standard management.^4^ Indeed, the definition of optimal PK/PD target attainment of beta-lactams is currently undergoing a paradigm shift in critically ill patients, because it is argued that targeting concentrations to higher thresholds may be of further benefit.^5^ Of note, a recent meta-analysis showed that aggressive PK/PD target attainment of traditional beta-lactams, defined as 100%fT_>4xMIC_, which is equivalent to a free (*f*) C_ss_ or *f*C_min_/minimum inhibitory concentration (MIC) ratio >4, performed better than the conservative PK/PD target of 100%fT_>MIC_, which is equivalent to an *f*C_ss_ or *f*C_min_/MIC ratio of 1, in the treatment of gram-negative infections. The aggressive target was associated with an improved clinical cure rate [odds ratio (OR): 1.69; 95% confidence interval (CI), 1.15–2.49] and a reduced risk of the development of beta-lactam resistance (OR: 0.06; 95% CI, 0.01–0.29).^6^

In 2021, at our tertiary university hospital, we established an innovative TDM-guided expert clinical pharmacological advice (ECPA) program for optimizing PK/PD target attainment and personalizing therapies with traditional antimicrobial agents in the critical care setting.^7^ The program was subsequently extended hospital-wide,^8^ and finally to novel BL/BLIc and cefiderocol treatments, given the worrisome increasing prevalence of DTR gram-negative–related infections at our hospital. This is in agreement with the recent recommendations of the French Intensive Care Society, suggesting that a TDM-guided approach using novel BL/BLIc and cefiderocol treatments may be especially helpful for properly rationalizing their use in critically ill patients, especially in the presence of certain risk factors for underexposure.^9^

The aim of this study was to perform an interim analysis of the findings of our program 3 years after starting it.

## MATERIALS AND METHODS

This retrospective observational study included patients receiving ceftazidime–avibactam, ceftolozane–tazobactam, meropenem–vaborbactam, and cefiderocol who underwent TDM-guided ECPAs from March 2021 to March 2024 at the IRCCS Azienda Ospedaliero-Universitaria of Bologna, a 1362-bed tertiary care Italian university hospital. Based on clinical needs, the program started with ceftazidime–avibactam and cefiderocol in March 2021 and was subsequently extended to meropenem–vaborbactam in March 2023 and finally to ceftolozane–tazobactam in November 2023.

The following demographic and clinical features were collected for each patient: age; sex; weight; height; body mass index (BMI); measured or estimated creatinine clearance according to 24-h urine collection or the Chronic Kidney Disease Epidemiology Consortium (CKD-EPI) formula, respectively; the need for continuous renal replacement therapy (CRRT); the occurrence of augmented renal clearance (ARC), the ward of hospital admission [intensive care unit (ICU), medicine, hematology, surgery, or pediatrics]; underlying diseases; the type of antibiotic treatment (ie, empirical or targeted mono or combo therapy); antibiotic dosing regimen and mode of administration [ie, intermittent infusion (II), extended infusion (EI) or continuous infusion (CI)]; the site of infection; gram-negative clinical isolates and susceptibility (MICs); the timing of TDM-guided ECPAs and the recommended dosing adjustments; aggressive PK/PD target attainment or nonattainment; microbiological eradication; clinical cure; and 30-day mortality. Microbiological eradication was defined as the eradication of the index pathogen from the infection site, as documented by follow-up cultures after more than 7 days from starting treatment with novel BL/BLIc or cefiderocol.^10^ Clinical cure was defined as the complete resolution of signs and symptoms of infection coupled with documented microbiological eradication at end of treatment and absence of recurrence/relapse at 30-day follow-up and/or attributable mortality due to infection.^10^ Whenever using CI, stability was achieved by reconstituting and administering the aqueous solutions based on previous findings (namely every 24 hours for ceftolozane–tazobactam and every 6–8 hours for ceftazidime–avibactam, cefiderocol, and meropenem–vaborbactam).^11^

Analytical methods for measuring the serum concentrations of novel antibiotics were established and validated using liquid chromatography tandem mass spectrometry. Briefly, for ceftazidime–avibactam, the method validated by Sillen et al^12^ was used, whereas new methods were developed and validated for the other antimicrobials.^13–15^ The lower limits of quantification were 0.04 and 0.01 mg/L for ceftazidime and avibactam, respectively^12^; 0.1 mg/L for cefiderocol,^13^ meropenem, and vaborbactam^14^; and 0.2 and 0.1 mg/L for ceftolozane and tazobactam, respectively.^15^ The intra- and interassay coefficients of variation were <15% for all methods.^12–15^

The first TDM assessment of the serum trough (C_min_) or steady-state concentration (C_ss_) was performed within 48–72 hours from the start of treatment and was subsequently reassessed whenever feasible on a case-by-case basis. TDM sessions were conducted twice or thrice weekly, with a turn-around-time of 48–72 hours. TDM-guided ECPAs were delivered to applicant clinicians, and dosing recommendations were provided for attaining aggressive PK/PD targets, as previously defined^16–19^ (Table 1). Briefly, for cefiderocol, the aggressive PK/PD target was defined as an *f*C_min_ or *f*C_ss_/MIC ratio equal to 4–8 (equivalent to 100%*f*T_>4-8 x MIC_ according to the time-dependent activity of cefiderocol).^19^ For BL/BLIc, a joint aggressive PK/PD target was selected. This was defined as a BL *f*C_min_ or *f*C_ss_/MIC ratio equal to 4–8 (equivalent to 100%*f*T_>4-8 x MIC_ according to the time-dependent activity of BL/BLIc) coupled with a BLI *f*C_min_ or *f*C_ss_/target concentration (C_T_) ratio of 1–4 for ceftazidime–avibactam or ceftolozane–tazobactam, or with a free area under the concentration-to-time curve (*f*AUC)/C_T_ ratio of 24–96 for meropenem–vaborbactam.^16–18^ Specifically, the AUC of vaborbactam was calculated using the following formula: AUC (mg*h/L) = dose (mg/24 hours)/clearance [CL] (L/h), where CL was calculated as the infusion rate (mg/h)/C_ss_ (mg/L). The free fractions were calculated by subtracting from the total concentration of each BL or BLI the specific protein bound rate, as reported in the literature, namely 2% for meropenem,^20^ 7% for avibactam,^21^ 10% for ceftazidime,^21^ 20% for ceftolozane,^22^ 23% for tazobactam,^22^ 33% for vaborbactam,^23^ and 58% for cefiderocol.^24^ The C_T_ corresponded to the fixed BLI target concentration defined by the European Committee on Antimicrobial Susceptibility Testing for testing the in vitro standard susceptibility of each BL/BLIc, namely, 4 mg/L for both tazobactam and avibactam, and 8 mg/L for vaborbactam.

**TABLE 1.** Desired PK/PD Targets, Toxicity Thresholds, and Recommended Dosing Adjustments for Novel Anti–Gram-negative Beta-Lactams

Novel Anti–Gram-negative Beta-Lactams	Targeted Indications	Aggressive PK/PD Target for Maximizing Efficacy	Exposure Threshold for Preventing Toxicity	Recommended Dosing Adjustment
Ceftazidime–avibactam	KPC- and/or OXA-48–producing *Enterobacterales* infections DTR *Pseudomonas aeruginosa* infections	Joint: Ceftazidime: C_ss_/MIC or C_min_/MIC = 4–8 plus avibactam C_ss_/C_T_ or C_min_/C_T_ > 1	Ceftazidime C_min_ > 104 mg/L (neurotoxicity)^25^ Avibactam NA	Decrease*: 50% if ceftazidime C_min_ or C_ss_ > 10 × MIC 25% if ceftazidime C_min_ or C_ss_ 8–10 × MIC and avibactam C_ss_/C_T_ or C_min_/C_T_ > 4 Increase: 50% if ceftazidime C_min_ or C_ss_ < 2 × MIC 25% if ceftazidime C_min_ or C_ss_ 2–4 × MIC and/or avibactam C_ss_/C_T_ or C_min_/C_T_ < 1
Ceftolozane–tazobactam	DTR *P. aeruginosa* infections ESBL-producing *Enterobacterales* infections	Ceftolozane C_ss_/MIC or C_min_/MIC = 4–8 Joint†: Ceftolozane: C_ss_/MIC or C_min_/MIC = 4–8 plus tazobactam C_ss_/C_T_ or C_min_/C_T_ > 1	NA	Decrease*: 50% if ceftolozane C_min_ or C_ss_ > 10 × MIC 25% if ceftolozane C_min_ or C_ss_ 8–10 × MIC and tazobactam C_ss_/C_T_ or C_min_/C_T_ > 4 Increase: 50% if ceftolozane C_min_ or C_ss_ < 2 × MIC 25% if ceftolozane C_min_ or C_ss_ 2–4 × MIC and/or tazobactam C_ss_/C_T_ or C_min_/C_T_ < 1
Meropenem–vaborbactam	KPC-producing *Enterobacterales* infections ESBL-producing *Enterobacterales* infections (for specific patients colonized by KPC-producing *Enterobacterales*)	Joint: Meropenem: C_ss_/MIC or C_min_/MIC = 4–8 plus vaborbactam AUC/MIC >24	Meropenem C_min_ > 64.2 mg/L (neurotoxicity)^26^ Vaborbactam NA	Decrease*: 50% if meropenem C_min_ or C_ss_ > 10 × MIC 25% if meropenem C_min_ or C_ss_ 8–10 × MIC and vaborbactam AUC/MIC >96 Increase: 50% if meropenem C_min_ or C_ss_ < 2 × MIC 25% if meropenem C_min_ or C_ss_ 2–4 × MIC and/or vaborbactam AUC/MIC <24
Cefiderocol	KPC-, OXA-48–, and/or MBL-producing *Enterobacterales* infections DTR *P. aeruginosa* infections CRAB infections	C_ss_/MIC or C_min_/MIC = 4–8	NA	Decrease: 50% if C_min_ or C_ss_ > 10 × MIC 25% if C_min_ or C_ss_ 8–10 × MIC Increase: 50% if C_min_ or C_ss_ < 2 × MIC 25% if C_min_ or C_ss_ 2–4 × MIC

* Greater dosing reduction are allowed if ceftazidime or meropenem concentrations are above the toxicity thresholds.

† Joint PK/PD target attainment required only for ESBL-producing *Enterobacterales* infections.

C_min_, trough concentration; CRAB, carbapenem-resistant *Acinetobacter baumannii*; C_ss_, steady-state concentration; C_T_, target concentration (=4 mg/L for avibactam and tazobactam according to European Committee on Antimicrobial Susceptibility Testing recommendations); KPC, *Klebsiella pneumoniae*–producing carbapenemase; MBL, metallo-beta-lactamase; MIC, minimum inhibitory concentration; NA, not assessed.

The BL/BLIc dosing recommendations were as previously described^7,18^ and are detailed in Table 1. Briefly, dosing was confirmed if the *f*C_ss_/MIC or *f*C_min_/MIC ratio of the specific BL was 4–8, and the *f*C_ss_/C_T_ or *f*C_min_/C_T_ ratio of avibactam or tazobactam was 1–4, or the *f*AUC/C_T_ ratio of vaborbactam was 24–96. A 25% or 50% dose decrease was adopted if the *f*C_ss_/MIC or *f*C_min_/MIC ratio of the specific BL was 8–10 or >10, respectively; the *f*C_ss_/C_T_ or *f*C_min_/C_T_ ratio of avibactam or tazobactam was >4; or the *f*AUC/C_T_ ratio of vaborbactam was >96. The upper threshold concentrations for preventing neurotoxicity with ceftazidime and meropenem were considered as 64.2 mg/L^25^ and 104 mg/L,^25^ respectively. A 25% or 50% dose increase was implemented if the *f*C_ss_/MIC or *f*C_min_/MIC ratio of the specific BL was 2–4 or below 2, respectively; the *f*C_ss_ or *f*C_min_/C_T_ ratio of avibactam or tazobactam was <1; or the *f*AUC/C_T_ ratio of vaborbactam was <24.

Three performance indicators were identified to assess the utility of the program in clinical need and to improve the likelihood of attaining aggressive PK/PD targets either early or during the overall treatment period. First, the average number of ECPAs delivered per month of program availability for each agent was considered as an indicator of the overall utility of the ECPA program in clinical need. Second, the ratio at the first TDM assessment between the total number of ECPAs recommending dosing adjustments and the total number of delivered ECPAs was assumed as an indicator of the utility of the ECPA program for allowing early optimization of PK/PD target attainment. Third, the ratio of subsequent TDM assessments between the total number of ECPAs recommending dosing adjustments and the total number of delivered ECPAs was assumed as an indicator of the performance of the ECPA program in allowing aggressive PK/PD target attainment during the overall treatment period.

The relationship between aggressive PK/PD target attainment or nonattainment and clinical or microbiological outcomes was identified as a potential additional indicator of program performance and assessed using the χ^2^ test or Fisher exact test.

Data are expressed as the mean ± SD or the median and interquartile range (IQR) or range according to the data distribution, whereas categorical variables are expressed as counts and percentages. A comparison of the proportions of ECPAs recommending dosing adjustments between the first and subsequent TDM assessments was conducted using χ^2^ or Fisher exact tests as appropriate for categorical variables. Statistical significance was set at *P* < 0.05.

## RESULTS

A total of 595 TDM-guided ECPAs were provided to 263 patients [mainly admitted to the ICU (56.3%)] to optimize 319 treatment courses (151 with ceftazidime–avibactam, 68 with cefiderocol, 56 with meropenem–vaborbactam, and 44 with ceftolozane–tazobactam). The demographic and clinical characteristics are reported in Table 2. The median (IQR) age was 64 years (53–74 years), with a male predominance (68.4%). Of the included patients, 13.3% were obese (BMI >30 kg/m^2^), 18.6% underwent CRRT, and 5.7% received ARC. The most frequent underlying diseases were hematological disorders/malignancies (19.1%), solid organ transplantation (15.2%), abdominal perforation (9.1%), and COVID-19–associated acute respiratory distress syndrome (9.1%).

**TABLE 2.** Demographics and Clinical Characteristics of Hospitalized Patients Undergoing a TDM-Guided Expert Clinical Pharmacological Advice Program for Novel Beta-Lactam/Beta-Lactamase Inhibitors and Cefiderocol

Patient Demographic	Overall (N = 263)	Ceftazidime–Avibactam (N = 119)	Cefiderocol (N = 53)	Meropenem–Vaborbactam (N = 47)	Ceftolozane–Tazobactam (N = 44)
Age (yr) [median (range)]	64 (1–92)	64 (1–92)	59 (1–81)	64 (30–88)	63.5 (15–90)
Sex (male/female) [n (%)]	180/83 (68.4/31.6)	80/39 (67.2/32.8)	41/12 (77.4/22.6)	33/14 (70.2/29.8)	26/18 (59.1/40.9)
Body weight (kg) [median (range)]	74.0 (2.8–130.0)	75 (9–122)	77.5 (2.8–100)	71.8 (48–130)	70 (47–119)
Body mass index (kg/m^2^) [median (range)]	24.9 (11.2–50.8)	24.7 (12.9–39.2)	25.2 (11.2–36.7)	24.2 (17.3–50.8)	25.5 (16.7–41.2)
Obesity (BMI >30 kg/m^2^) [n (%)]	35 (13.3)	19 (16.0)	3 (5.7)	5 (10.6)	8 (18.2)
CL_CR_ (mL/min/1.73 m^2^) [median (range)]	90 (1.5–257)	94 (3.9–257)	84 (8–169)	84 (1.5–131)	55 (10–136)
Augmented renal clearance* [n (%)]	15 (5.7)	9 (7.6)	3 (5.7)	2 (4.3)	1 (2.3)
CRRT [n (%)]	49 (18.6)	21 (17.6)	9 (17.0)	11 (23.4)	8 (18.2)
Setting [n (%)]					
ICU	148 (56.3)	49 (41.2)	37 (69.8)	32 (68.1)	30 (68.2)
Medicine	63 (24.0)	39 (32.8)	9 (17.0)	8 (17.0)	7 (15.9)
Hematology	25 (9.5)	16 (13.4)	2 (3.8)	3 (6.4)	4 (9.1)
Surgery	18 (6.8)	11 (9.2)	1 (1.9)	4 (8.5)	2 (4.5)
Pediatrics	9 (3.4)	4 (3.4)	4 (7.5)	0 (0.0)	1 (2.3)
Underlying disease [n (%)]					
Hematological disorders/malignancies	50 (19.1)	32 (26.9)	6 (11.3)	5 (10.6)	7 (15.8)
Solid organ transplant	40 (15.2)	14 (11.7)	5 (9.4)	13 (27.7)	8 (18.2)
Abdominal perforation	24 (9.1)	12 (10.1)	3 (5.7)	8 (17.0)	1 (2.3)
ARDS associated with COVID-19	24 (9.1)	8 (6.7)	15 (28.3)	0 (0.0)	1 (2.3)
Complications after cardiosurgical intervention	19 (7.2)	7 (5.9)	1 (1.9)	2 (4.3)	9 (20.5)
Complications after orthopedic intervention	15 (5.7)	8 (6.7)	6 (11.3)	1 (2.1)	0 (0.0)
Acute respiratory failure	12 (4.6)	4 (3.4)	0 (0.0)	4 (8.5)	4 (9.1)
ACLF	9 (3.4)	5 (4.2)	1 (1.9)	1 (2.1)	2 (4.5)
Cholecystitis/cholangitis	9 (3.4)	6 (5.0)	2 (3.8)	1 (2.1)	0 (0.0)
Solid cancer	9 (3.4)	4 (3.4)	2 (3.8)	3 (6.4)	0 (0.0)
Acute pancreatitis	4 (1.5)	0 (0.0)	0 (0.0)	3 (6.4)	1 (2.3)
Other	48 (18.3)	19 (16.0)	12 (22.6)	6 (12.8)	11 (25.0)
Antibiotic treatment [n (%)]					
Treatment courses	319	151	68	56	44
Empirical	65 (20.4)	39 (25.8)	4 (5.9)	15 (26.8)	7 (15.9)
Targeted	254 (79.6)	112 (74.2)	64 (94.1)	41 (73.2)	37 (84.1)
Continuous infusion	264 (82.8)	143 (94.7)	34 (50.0)	44 (78.6)	43 (97.7)
Extended/intermittent infusion	55 (17.2)	8 (5.3)	34 (50.0)	12 (21.4)	1 (2.3)
Mono vs. combination therapy	211/108 (66.1/33.9)	94/57 (62.3/37.7)	28/40 (41.2/58.8)	53/3 (94.6/5.4)	36/8 (81.8/18.2)
Aggressive PK/PD target attainment	267/319 (83.7)	118/151 (78.1)	58/68 (85.3)	47/56 (83.9)	44/44 (100.0)
Clinical outcome [n (%)]					
Clinical cure	203/319 (63.6)	107/151 (70.9)	35/68 (51.5)	35/56 (62.5)	26/44 (59.1)
Microbiological eradication	159/208 (76.4)	80/93 (86.0)	32/53 (60.4)	24/32 (75.0)	23/30 (76.7)
30-day mortality rate	75 (23.5)	29 (19.2)	24 (35.3)	14 (25.0)	8 (18.2)

Data are presented as the median (range) for continuous variables and as n (%) for dichotomous variables.

* Augmented renal clearance was defined as a normal serum creatinine level coupled with an estimated CL_CR_ > 130 mL/min/1.73 m^2^ in males or >120 mL/min/1.73 m^2^ in females.^27^

ACLF, acute-on-chronic liver failure; ARDS, acute respiratory distress syndrome; CL_CR_, creatinine clearance.

Novel agents were administered mainly via CI (82.8%, ranging from 50.0% with cefiderocol to 97.7% with ceftolozane–tazobactam), targeted treatment of microbiologically documented gram-negative infections (254/319; 79.6%, ranging from 73.2% with meropenem–vaborbactam to 94.1% with cefiderocol), and monotherapy (66.1%, ranging from 50.0% with cefiderocol to 97.7% with ceftolozane–tazobactam; see **Figure**, **Supplemental Digital Content 1**, http://links.lww.com/TDM/A837).

**FIGURE 1.**
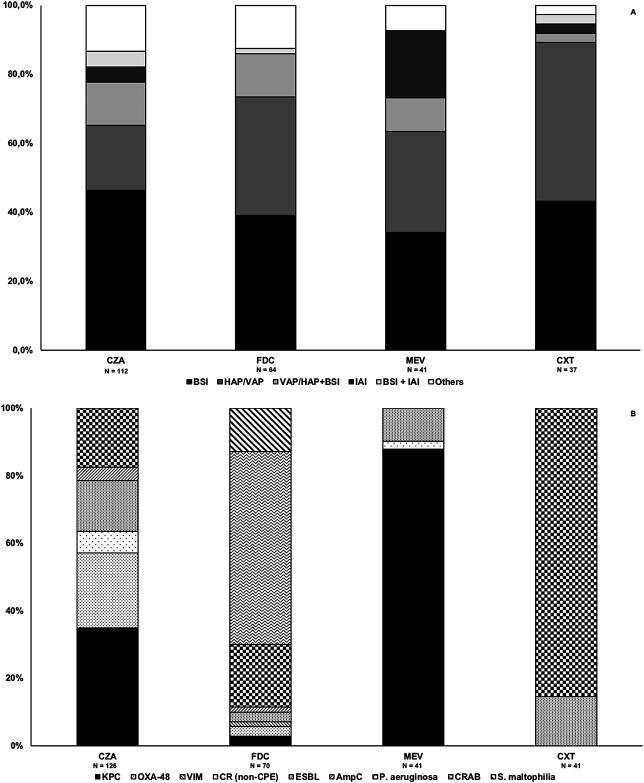
Types of infections and isolated pathogens in patients receiving targeted therapies with novel beta-lactam/beta-lactamase inhibitors and cefiderocol. A, Proportion of the types of infection for targeted therapies with novel beta-lactam/beta-lactamase inhibitors and cefiderocol. Other infections: ceftazidime–avibactam: five bone and joint infections (BJIs); four urinary tract infections (UTIs); two BJIs plus BSIs; two skin and soft tissue infections (SSTIs); one central nervous system (CNS) infection; one CNS infection plus BSI; cefiderocol: four BJIs; one BJI plus BSI; one CNS infection; one CNS infection plus BSI; one SSTI; meropenem–vaborbactam: three ventilator-associated pneumonia (VAPs) plus intra-abdominal infections (IAIs); one UTI; ceftolozane–tazobactam: one UTI; (B) proportion of isolated pathogens for targeted therapies with novel beta-lactam/beta-lactamase inhibitors and cefiderocol.

Overall, targeted therapies were mainly used for bloodstream infections (42.1% overall, 46.3% for ceftazidime–avibactam, 39.0% for cefiderocol, and 34.2% for meropenem–vaborbactam), nosocomial pneumonia (28.3% overall, 45.9% for ceftolozane–tazobactam), and bacteremic pneumonia (10.6%; Fig. 1A). Ceftazidime–avibactam was used mainly for treating KPC- (34.9%) or OXA-48–producing (22.2%) *Enterobacterales*-related infections, cefiderocol was used mainly for treating carbapenem-resistant *Acinetobacter baumannii* (57.1%) or DTR *Pseudomonas aeruginosa*–related infections (18.6%), meropenem–vaborbactam was used mainly for treating KPC- (87.8%) or extended-spectrum beta-lactamase (ESBL)-producing (9.8%) *Enterobacterales-*related infections, and ceftolozane–tazobactam was used mainly for treating DTR *P. aeruginosa* (85.4%) or ESBL-producing (14.6%) infections (Fig. 1B).

Aggressive PK/PD targets were attained in 83.7% of all treatment courses (267/319) (ranging from 78.1% for ceftazidime–avibactam to 100% for ceftolozane–tazobactam), and a clinical cure was achieved in 63.6% (203/319) of all treatment courses (ranging from 51.5% for cefiderocol to 70.9% for ceftazidime–avibactam). Microbiological outcomes were assessable in 208 of the 254 targeted treatment courses (81.9%), and eradication was achieved in 159 (76.4%). The overall 30-day mortality rate was 23.5% (of 75/319).

The features of the delivered TDM-guided ECPAs (n = 595) are summarized in **Supplemental Digital Content 3** (see **Table**, http://links.lww.com/TDM/A839). Most of the ECPAs were for ceftazidime–avibactam (282/595; 47.5%), followed by meropenem–vaborbactam (121/595; 20.3%), cefiderocol (115/595; 19.3%), and ceftolozane–tazobactam (77/595; 12.9%). Most of these drugs were administered to patients admitted to the ICU (51.8%, 51.9%, 70.4%, and 74.0% for ceftazidime–avibactam, meropenem–vaborbactam, cefiderocol, and ceftolozane–tazobactam, respectively). The average number of ECPAs delivered per month for program availability of each drug is shown in **Supplemental Digital Content 2** (see **Figure**, http://links.lww.com/TDM/A838). It was highest for ceftolozane–tazobactam (15.4 per month), followed by meropenem–vaborbactam (10.1 per month), ceftazidime–avibactam (7.8 per month), and cefiderocol (3.2 per month). The monthly distribution of the number of ECPAs delivered for each agent showed an overall increase in clinical requirements over time.

**FIGURE 2.**
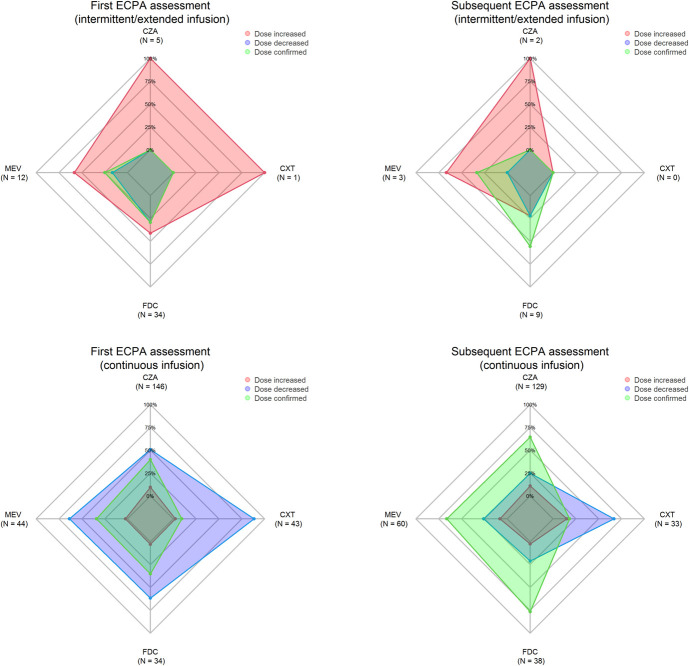
Radar charts of the proportion of dosing recommendations needed at the first and subsequent therapeutic drug monitoring assessments in relation to intermittent/extended infusion or continuous infusion administration of each agent.

Radar charts showing the proportion of dosing recommendations provided during the first and subsequent TDM assessments in relation to II/EI or CI administration are shown for each agent in Figure 2. Overall, at the first TDM assessment, the proportion of ECPAs recommending dosing adjustments was higher in the II/EI group than the CI group (75.0% vs. 66.7%, *P* = 0.24). Dose increases were mostly required for patients receiving II/EI administration (51.9% vs. 6.4%; *P* < 0.0001), whereas dose decreases were mostly recommended for patients receiving CI administration (60.3% vs. 23.1%; *P* < 0.001). Regarding the status of the patients' renal function, patients with ARC required significantly more frequent dosing increases than those with sepsis-related acute kidney injury (AKI; 62.5% vs. 10.5%; *P* = 0.003) or chronic kidney disease (CKD; 62.5% vs. 3.2%; *P* < 0.001), whereas the opposite was true for dosing decreases [6.3% for patients with ARC compared with 78.9% in those with sepsis-related AKI (*P* < 0.001) and 74.2% for those with CKD (*P* < 0.001)].

In the subsequent TDM assessments, the overall proportion of ECPAs recommending dosing adjustments decreased in both groups (57.1% of patients receiving II/EI administration and 39.3% of those receiving CI administration). The dosing adjustment tended to a higher proportion of increases in the II/EI group (increase: 42.9%, decrease: 14.2%; *P* = 0.19) and of decreases in the CI group (decrease: 29.8%, increase: 9.5%; *P* < 0.0001). In patients receiving CI administration, the need for dosing adjustments decreased significantly for ceftazidime–avibactam (60.3% vs. 35.7%, *P* < 0.0001), meropenem–vaborbactam (65.9% vs. 33.9%, *P* = 0.001), and cefiderocol (64.7% vs. 23.7%, *P* = 0.0005), but remained high for ceftolozane–tazobactam (90.7% vs. 81.9%, *P* = 0.32).

The relationships between aggressive PK/PD target attainment or nonattainment of each agent, microbiological outcomes, and clinical outcomes are summarized in Table 3. Aggressive PK/PD target attainment of ceftazidime–avibactam was significantly associated with the highest microbiological eradication rate (79.6% of assessable microbiological outcomes; 74/93; *P* < 0.001) and clinical cure rate (64.2% of assessable clinical outcomes; 97/151; *P* < 0.001). Likewise, aggressive PK/PD target attainment with cefiderocol was associated with the highest proportion of clinical cure rates (50.0% of assessable clinical outcomes; 34/68; *P* = 0.006). Conversely, no relationship was observed between aggressive PK/PD target attainment of meropenem–vaborbactam and ceftolozane–tazobactam and microbiological/clinical outcomes.

**TABLE 3.** Relationship Between Aggressive PK/PD Target Attainment or Nonattainment of Each Novel Antibiotic and the Microbiological or Clinical Outcome of the Treatment Courses

Novel Agent	Microbiological Outcome		*P*	Clinical Outcome		*P*
	Microbiological Eradication	Microbiological Failure		Clinical Cure	Clinical Failure	
Ceftazidime–avibactam						
Aggressive PK/PD target attainment	74/93 (79.6%)	4/93 (4.3%)	**< 0.001**	97/151 (64.2%)	21/151 (13.9%)	**< 0.001**
Aggressive PK/PD target nonattainment	6/93 (6.4%)	9/93 (9.7%)		10/151 (6.6%)	23/151 (15.3%)	
Cefiderocol						
Aggressive PK/PD target attainment	28/53 (52.8%)	15/53 (28.3%)	0.17	34/68 (50.0%)	24/68 (35.3%)	**0.006**
Aggressive PK/PD target nonattainment	4/53 (7.5%)	6/53 (11.3%)		1/68 (1.5%)	9/68 (13.2%)	
*Meropenem–vaborbactam*						
Aggressive PK/PD target attainment	20/32 (62.5%)	7/32 (21.9%)	0.99	27/56 (48.2%)	20/56 (35.7%)	0.13
Aggressive PK/PD target nonattainment	4/32 (12.5%)	1/32 (3.1%)		8/56 (14.3%)	1/56 (1.8%)	
Ceftolozane–tazobactam						
Aggressive PK/PD target attainment	23/30 (76.7%)	7/30 (23.3%)	0.99	26/44 (59.1%)	18/44 (40.9%)	0.99
Aggressive PK/PD target nonattainment	0/30 (0.0%)	0/30 (0.0%)		0/44 (0.0%)	0/44 (0.0%)	

Values in bold are statistically significant

## DISCUSSION

To the best of our knowledge, this is the first study to assess the utility of a TDM-guided ECPA program for ceftazidime–avibactam, meropenem–vaborbactam, ceftolozane–tazobactam, and cefiderocol in hospitalized patients with documented or suspected gram-negative DTR infections. Overall, the findings may support the contention that such a program, by addressing different unanswered issues linked to the use of novel BL/BLIc and cefiderocol, may be especially helpful in hospital settings, such as ours, which are burdened by case-mix complexity, with a high prevalence of DTR gram-negative pathogens.

Our program, which maximizes the likelihood of attaining aggressive PK/PD targets of novel BLs, is in line with the recent recommendations of the French Intensive Care Society dealing with an unmet need to appropriately rationalize the use of new BL/BLIc drugs and cefiderocol in critically ill patients.^9^

First, ceftazidime–avibactam, meropenem–vaborbactam, ceftolozane–tazobactam, and cefiderocol were mainly used as targeted monotherapies for documented DTR gram-negative infections in critically ill patients and were administered mainly via CI. Prolonged infusion of traditional BLs in critically ill patients was associated with either an improved likelihood of PK/PD target attainment or better clinical and/or microbiological cure rates compared with II in two recent meta-analyses.^28,29^ Although prolonged infusion encompasses both EI and CI, it is worth noting that CI should be considered superior to EI in certain challenging settings.^30^ CI administration has been recently recommended for new BLs, especially when dealing with clinical scenarios of critically ill patients with a high risk of suboptimal PK/PD target attainment, namely in presence of ARC, for pathogens with a high MIC, and/or for deep-seated infections with a low penetration rate.^6,31^ Of note, Pilmis et al^32^ showed prospectively that administering 3 g q8h of ceftolozane–tazobactam by CI provided a markedly higher probability of attaining an aggressive PK/PD target compared with both EI and II when dealing with DTR *P. aeruginosa* infections caused by strains with borderline susceptibility to ceftolozane–tazobactam (MICs ≥4 mg/L). Likewise, a 7-day hollow-fiber infection model compared the efficacy of three different dosages of ceftolozane–tazobactam (3g q8h by II [over 1 hour], 3 g q8h by EI [over 4 hours], and 6 g by CI) against three extensively drug resistant (XDR) *P. aeruginosa* isolates (one susceptible [MIC of 2 mg/L] and two resistant [MIC of 8 and 16 mg/L] to ceftolozane–tazobactam).^33^ CI of the lower dosage resulted in aggressive PK/PD target attainment against all isolates and the largest decrease in the total number of bacterial colonies in all treatment arms and was the only bactericidal regimen against all isolates. This approach should be considered a potential treatment strategy for infections caused by XDR *P. aeruginosa* isolates, including in vitro nonsusceptible isolates.^33^

The utility in the clinical need that such a program may have in clinical settings like ours is supported by the findings for all three identified performance indicators. First, most TDM-guided ECPAs were delivered to patients in the ICU, which is in agreement with DTR gram-negative–related infections being highly prevalent among critically ill patients.^34^ The total number of ECPAs delivered increased over time, and the average number of ECPAs delivered per month of program availability varied consistently among the four agents. This suggests that the clinical needs for the four agents varied over time. The highest was observed for ceftolozane–tazobactam, which is consistent with DTR-*P. aeruginosa* being the most challenging pathogen currently encountered in our clinical setting. This was quite high for both meropenem–vaborbactam and ceftazidime–avibactam, as KPC- and OXA-48–producing *Enterobacterales* were prevalent in our hospital and caused different outbreaks over time. Conversely, it was more limited for cefiderocol, because of clusters of carbapenem-resistant *A. baumannii*–related infections occurring especially in the first year of the COVID-19 pandemic.^19,35^ Second, the prevalent need of dosing adjustments at the first TDM assessment was affected by the mode of drug administration. Dose increases were mainly required for patients receiving novel agents via II or EI. This highlights the difficulty of attaining aggressive PK/PD targets with such administration modalities, a condition that might increase the risk of microbiological failure and the development of resistance.^6,31,36^ Conversely, dosing decreases were mostly recommended for patients receiving novel agents by CI. This can be explained by two factors. The availability of such a program facilitated the recently recommended approach of administering full dosages during the first 24–48 hours of treatment, even for patients with sepsis-associated AKI.^9^ This may prevent the risk of suboptimal PK/PD target attainment in cases of transient reversal AKI, leading to rapid recovery of renal function.^37,38^ In addition, CI allowed aggressive PK/PD target attainment with lower dosages when dealing with targeted therapies against clinical isolates with low MIC values.^7^ Third, the consistent decrease in the proportion of ECPAs recommending dosing adjustments at subsequent TDM assessments demonstrated the high compliance of clinicians in applying the recommendations, and this may further support the usefulness of establishing such a program.

Our interim analysis showed that aggressive joint PK/PD target attainment of ceftazidime/avibactam and cefiderocol may explain most of the microbiological eradication and/or clinical cure rates obtained with these agents. These promising findings may have implications in clinical practice, considering that the emergence of breakthrough resistance to novel BL/BLIc and cefiderocol has recently been reported and that some recent epidemiological studies have shown worrisome increasing rates of resistance.^39–42^ Tailoring treatment using a TDM-based ECPA program and maximizing the likelihood of attaining aggressive PK/PD targets with novel BL/BLIc may represent a cost-effective strategy either for preventing the development of resistance or for reducing the need for combination therapy. In this regard, we recently showed that attaining an aggressive joint PK/PD target with CI ceftazidime–avibactam monotherapy allowed microbiological eradication of deep-seated DTR gram-negative infections and may make combination therapy unnecessary.^10^

Our study has some limitations. The retrospective study design is acknowledged. Imipenem–relebactam was not included in the program because its use was limited in our hospital. We recognize that our data analysis was mainly descriptive, and that including a control group with no TDM would have allowed us to test the real added value of the TDM-guided approach. The aggressive PK/PD targets used in this study were selected based on previous studies,^10,17,19^ and we recognize that establishing specific PK/PD target–outcome relationships in this population would have been valuable. The use of combination therapy is a potential confounder in the assessment of clinical and microbiological outcomes. Finally, we recognize that assessing the impact of different modes of drug administration on the emergence of resistance may have been interesting, even if this purpose was outside the scope of our study.

## CONCLUSIONS

In conclusion, the findings of this study support the contention that establishing a TDM-guided ECPA program for ceftazidime–avibactam, meropenem–vaborbactam, ceftolozane–tazobactam, and cefiderocol for attaining aggressive PK/PD targets may be helpful in hospital settings with case-mix complexity burdened with a high prevalence of DTR gram-negative pathogens and may result in better microbiological/clinical outcomes. Prospective confirmatory studies are warranted to demonstrate the positive impact of such a program on the clinical and microbiological outcomes of DTR-gram negative infections.

## Supplementary Material

**Figure:**
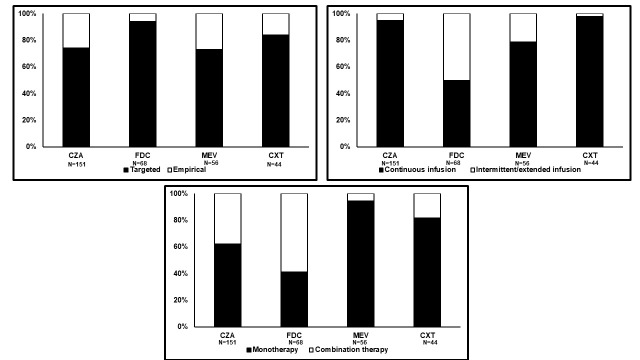


**Figure:**