# Supplementary material for: Utility of a TDM-Guided Expert Clinical Pharmacological Advice Program for Optimizing the Use of Novel Beta-Lactam/Beta-Lactamase Inhibitor Combinations and Cefiderocol in a Tertiary University Hospital: An Interim Analysis
Source: Ther Drug Monit. 2025 May 2;47(6):809–19. doi: 10.1097/FTD.0000000000001334 (PMC12588651; doi:10.1097/FTD.0000000000001334)
Supplement: Supplementary file 1 [file tdm-47-0809-s003.docx]

**Supplementary materials**

**Supplementary Table 1 –** Features of the delivered TDM-guided ECPAs

**Supplementary Figure 1 –** Proportion of targeted vs. empirical therapy, continuous vs. intermittent/extended infusion, and mono vs. combo therapy for treatment courses adopted with novel beta-lactam/beta-lactamase inhibitors and cefiderocol

**Supplementary Figure 2 –** Monthly distribution over time of the absolute number of delivered ECPAs for each agent

| **Supplementary Table 1 –** Features of the delivered TDM-guided ECPAs | | | | | | | | |
| --- | --- | --- | --- | --- | --- | --- | --- | --- |
| **Novel beta-lactam** | **No. of ECPAs** | **No. of treatment courses** | **No. of ECPAs per month of program availability** | **Hospital settings** | | | | |
|  |  |  |  | **ICU** | **Medicine** | **Hematology** | **Surgery** | **Pediatrics** |
| Ceftazidime-avibactam | 282 | 151 | 7.8 | 146  (51.8%) | 56  (19.8%) | 31  (11.0%) | 35  (12.4%) | 14  (5.0%) |
| Meropenem-vaborbactam | 121 | 56 | 10.1 | 87  (71.9%) | 11  (9.1%) | 12  (9.9%) | 11  (9.1%) | 0  (0.0%) |
| Cefiderocol | 115 | 68 | 3.2 | 81  (70.4%) | 17  (14.8%) | 7  (6.1%) | 1  (0.9%) | 9  (7.8%) |
| Ceftolozane-tazobactam | 77 | 44 | 15.4 | 57  (74.0%) | 13  (16.9%) | 4  (5.2%) | 2  (2.6%) | 1  (1.3%) |
| ECPA: expert clinical pharmacology advice; ICU: intensive care unit; IQR interquartile range | | | | | | | | |
